# Manganese-Exchanged Smectite Clays as a Potential Controlled-Release Fertilizer for Mn Nutrition: Comparative Evaluation of Mn-Laponite and Mn-Montmorillonite

**DOI:** 10.1021/acs.jafc.6c03852

**Published:** 2026-07-24

**Authors:** María del Carmen García-Rico, Juan J. Lucena, Ana Carmen Perdigón Aller, Rosa Martín-Rodríguez, Sandra López-Rayo

**Affiliations:** † Department of Agricultural Chemistry and Food Science. Faculty of Science, Universidad Autónoma de Madrid, Avenida Francisco Tomás y Valiente, 7, 28049 Madrid, Spain; ‡ QUIPRE Department. ETSIIT, Universidad de Cantabria, Avenida de Los Castros, 46, 39005 Santander, Spain; § Nanomedicine Group. IDIVAL, Avenida Cardenal Herrera Oria s/n, 39011 Santander, Spain

**Keywords:** manganese, clays, controlled-release fertilizers, barley

## Abstract

To mitigate the negative effects of conventional fertilization, a novel concept of clay-based controlled-release manganese (Mn) fertilizers was investigated. Three Mn-exchanged smectites were synthesized via cation exchange: one montmorillonite (Mn­(NO_3_)_2_) and two laponites (Mn­(NO_3_)_2_ and MnCl_2_). Their composition, stability, and Mn-release behavior were assessed in saline medium across pH 5.5–8.2. A hydroponic experiment with barley (*Hordeum vulgare* cv. Antonia), a Mn-sensitive species, was conducted at pH 8.2 using sand amended with Mn-clays and compared with MnSO_4_ and MnEDTA. All treatments rapidly corrected Mn deficiency. At 8 weeks, biomass under Mn–laponite2, Mn–montmorillonite, and conventional fertilizers was comparable to or higher than that of the deficient control. Mn–laponites provided rapid Mn availability, whereas Mn–montmorillonite exhibited slower, sustained release. Smectite type thus controls Mn-release dynamics, supporting the use of synthetic clays as fertilizers in calcareous systems, though field validation is still pending.

## Graphical abstract

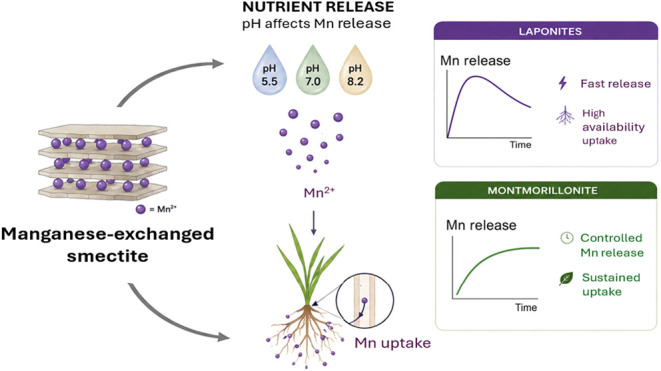

## 1 Introduction

Manganese is an essential element for normal plant growth and development, as it acts as an enzyme activator, often enhanced by other metal ions.^1^ Manganese is involved in activation reactions of catalytic enzymes, which include phosphorylation, decarboxylation, reduction, and hydrolysis reactions, thus affecting respiration processes, amino acid synthesis, lignin biosynthesis, and the level of plant hormones.^1–4^

In the absence of Mn, symptoms such as reduced yield and growth, as well as leaf yellowing, can be observed due to decreased chlorophyll levels, leading to interveinal chlorosis and necrosis in young leaves.^5^ The problem of Mn deficiency is especially relevant in areas with limestone soils,^3^ which, together with a high organic matter content, favor the oxidation of Mn^2+^ to MnO_2_, making this chemical form unavailable to plants.^6^ Multiple factors, including pH and redox potential, influence the availability of soil Mn to plants. The Mn solubility is higher in acid soils and high redox potential, where Mn^2+^ predominates in the soil solution, and represents the most plant-available Mn form.^1^ Environmental conditions also affect Mn solubility in soil; greater availability occurs in hot, dry seasons and under waterlogging in acid soils, both of which are directly related to redox potential, as well as the inhibition of Mn-oxidizing organisms, which allows the chemical reduction of Mn oxides in these soils.^7^ This process particularly affects winter cereal crops, such as barley and wheat.^8^ Manganese deficiency can result in the total loss of crops,^9^ as there are no obvious visual symptoms, leading to an underestimation of the true magnitude of the deficiency during the growth phase.^10^ Consequently, ensuring an adequate and sustained Mn supply remains a major challenge for crop production, particularly in alkaline and calcareous soils.

To address nutritional deficiencies in established crops, fertilizer application remains necessary. However, under alkaline soil conditions, the efficiency of conventional Mn fertilizers has been demonstrated to be particularly limited. Several studies have shown that after the application of traditional Mn fertilizers (e.g., Mn sulfate, Mn chloride, Mn nitrate^11^.) in soils, only a small fraction of the supplied nutrient reaches the destination site, while the rest can be subjected to processes of adsorption, desorption, escape or filtration due to the imbalance between the rate of release of nutrients from fertilizers and the rate of absorption of nutrients by the roots, making them less efficient.^12^ This fact entails frequent fertilizer application, which can lead to problems such as overdose,^13^ weakening, and contamination of both the soil and the groundwater.^14^

Synthetic polyaminocarboxylic Mn chelates, such as ethylenediaminetetraacetic acid (EDTA), are widely used in agronomic practices because they maintain Mn in soluble, plant-available forms, reducing fixation and precipitation as compared to inorganic salts. However, studies in alkaline soils have demonstrated that chelates are neither sufficiently stable to provide Mn at adequate levels for plants, making it necessary to explore other compounds that are less reactive under these soil conditions.^15^ Adding manganese to soils does not necessarily increase plant-available manganese, as higher application rates may cause it to be converted into unavailable fractions.^16^ Moreover, chelates degrade slowly and persist in the environment, being susceptible to leaching into groundwater and causing environmental damage.^17^

For this reason, the application of controlled-release fertilizers (CRFs) could mitigate the negative effects of conventional fertilizers. CRFs represent an advanced way of applying nutrients to crops, as they deliver nutrients gradually.^18^ The efficiency of fertilizer use can be improved by applying CRFs, which provide greater control and more precise regulation of nutrient release times, thereby reducing the need for constant fertilizer application.^20^ The issue with these fertilizers is that their release rhythm, pattern, and duration are poorly regulated, as soil conditions can alter them. Nevertheless, they are characterized by their ability to improve plant growth conditions, reduce stress, and mitigate the specific toxicity of excessive nutrient supply in root areas.^19^ Likewise, CRFs increase nutrient availability by releasing nutrients slowly in the soil, thereby overcoming fixation processes.^18^ From an environmental perspective, CRFs reduce losses of supernatant nutrients to the environment.^19^ Consequently, fertilizer accumulation in the soil is minimized, thereby reducing eutrophication. Therefore, the development of controlled-release systems with predictable nutrient release behavior under specific soil conditions remains a key challenge.

Recent studies have proposed novel Mn fertilizer formulations aiming to improve Mn availability and use efficiency under challenging soil conditions. For instance, Kumar et al.^20^ developed a manganese-based nanoclay–polymer composite combined with nano-MnO_2_, which showed enhanced Mn availability and positive effects on wheat growth. Other nanomicronutrient fertilizers have been developed, showing a better synchronized micronutrient release with crop demand, potentially increasing yields and nutrient density while reducing inputs and environmental risks.^21,22^ These approaches highlight the growing interest in nanostructured and solid-phase Mn fertilizers; however, further research is required to understand better the mechanisms governing Mn release, stability, and bioavailability under alkaline soil conditions.

Clays have been widely described as a type of slow release fertilizer, and modified clays are among the most commonly used nanostructured materials for producing CRFs.^23^ They are predominantly phyllosilicates whose physicochemical properties depend on their layered structure and extremely fine particle size (typically <2 μm).^24^ These materials are also valued for their high resistance to weathering, purity, ease of application, and low cost.^25^ Furthermore, their large specific surface area, combined with characteristics such as chemical and mechanical stability, lamellar morphology, and high cation exchange capacity (CEC), makes clays excellent candidates for the development of new fertilizer systems.^26^ These attributes enable clays to function as efficient nutrient carriers, facilitating gradual nutrient release into the soil and reducing losses through leaching or volatilization. Additionally, surface modification, via ion exchange, intercalation or functionalization, can significantly enhance their ability to retain and release agrochemicals in a controlled manner.^27^ As a result, clay based nanostructures are increasingly recognized as sustainable alternatives to conventional fertilizers, contributing to improved nutrient use efficiency and reduced environmental impact.^28^ Among the different CRF strategies, clay-based systems offer unique opportunities due to their structural versatility and ion-exchange properties, making them especially attractive for micronutrient delivery.

The catalytic properties of clays can be modified by acid and salt treatments, conferring slow-release properties on fertilizers and improving soil water retention capacity.^29^ That is why knowledge of clay modification methods and the properties of modified clays can facilitate the development of agricultural management systems that guarantee the long-term sustainability of soil resources.^29^ Clays such as montmorillonite and laponite have mainly been explored as fertilizer carriers and soil amendments to improve nutrient use efficiency in agriculture. Most studies have focused on their application for the supply of nitrogen and phosphate, primary macronutrients essential for plant growth, often through controlled or slow-release formulations designed to reduce nutrient losses and environmental impacts.^30–33^ Additionally, these clay-based systems have been investigated as matrices for the controlled release of various organic agrochemicals, including herbicides and pesticides, providing benefits to both crop nutrition and protection simultaneously.^34,35^

Despite these advances, most clay-based fertilizer systems have been developed for macronutrients, while significantly less attention has been devoted to micronutrient delivery.

In contrast, there is comparatively little information on the use of clays as carriers for micronutrients, even though plants require these elements in much smaller quantities. Existing studies on clays as micronutrient fertilizers have mainly focused on smectitic clays, particularly modified montmorillonites, and on zeolitic materials as carriers for transition metals such as Fe, Mn, Zn, and, to a lesser extent, B. Early greenhouse experiments in tomato plants grown on deficient sandy soil compared micronutrients supplied as sulfates, EDTA chelates or attached to modified montmorillonite, and showed that Cu-, Zn- and Mn-loaded montmorillonite provided plant yields and tissue concentrations comparable to, or better than, conventional chelates and generally superior to simple salts, demonstrating its suitability as a solid-phase micronutrient carrier in plant efficacy studies.^36^ More recent work has examined Zn-exchanged montmorillonite as a slow-release Zn nanofertilizer, combining incubation tests to characterize Zn release kinetics with pot experiments in rice that evidenced improved growth, yield, and grain Zn enrichment relative to ZnSO_4_, thus coupling release behavior with plant response.^37^ Beyond smectites, zeolite-based formulations enriched with exchangeable Zn^2+^ have been developed and characterized through sorption–desorption and leaching assays, and subsequently tested as foliar fertilizers, where they showed more gradual Zn release and greater resistance to rain-induced losses than soluble ZnSO_4_, indicating their potential as controlled-release micronutrient sources.^38^ Most of the above-mentioned works have focused on enriched formulations, in which micronutrients are added as soluble salts or simple admixtures to clay-based matrices, without inducing substantial changes in the clay crystal structure. In these systems, the clay mainly acts as an inert carrier or a sorbent phase controlling nutrient adsorption–desorption and diffusion, rather than as a chemically tailored host. By contrast, only a limited number of studies have deliberately modified the lamellar structure of clays to incorporate micronutrients (e.g., Fe, Mn, Zn, B) into the interlayer or structural positions. In particular, Imran et al. (2016)^39^ and López-Rayo et al. (2017)^40^ chemically modified layered double hydroxides (LDH-type clays) to host Zn within their brucite-like layers, achieving promising results in Zn delivery in solution and to barley plants grown in sand media. This imbalance in the literature, with extensive attention to macronutrients but scarce data on micronutrient delivery, highlights an important research opportunity.

The objective of this study was to design Mn-smectitic clays with the potential to function as controlled-release Mn fertilizers under alkaline soil conditions. To achieve this, three Mn- exchanged clays were synthesized, two laponites and one montmorillonite, subjected to comprehensive chemical characterization, and evaluated for their effectiveness as controlled-release fertilizers. This evaluation involved assessing their stability and cation-exchange capacity in calcium and low-salinity solutions, and testing their efficacy as a Mn source for plant growth in a sand-based medium under high pH conditions. The hypotheses of this work were: (1) Mn-exchangable clays can provide a controlled release of Mn, ensuring prolonged availability of Mn to plants under alkaline conditions, (2) the stability and exchange capacity of the Mn-exhanged clays will be sufficient to maintain Mn release in Ca-rich and low-salinity environments, (3) Mn-exchanged clays will improve plant growth by supplying adequate Mn in high pH media, simulating alkaline soil environments, and (4) the behavior of the Mn-exchanged clays will be different depending on the smectite type evaluate, Mn-laponites and Mn-montmorillonite.

## 2 Materials and Methods

### 2.1 Manganese-Based Clays Preparation

Manganese–based clays were prepared by incorporating Mn via cation exchange into montmorillonite and laponite structures to evaluate the capacity of natural and synthetic smectites to host Mn in the interlayer and to assess the potential influence of the counterion on Mn behavior. Specifically, a natural dioctahedral smectice, montmorillonite from Wyoming, with the chemical formula (Ca_0.12_Na_0.32_K_0.05_)­[Al_3.01_Fe_0.41_^3+^Mn_0.01_Mg_0.54_Ti_0.02_]­[Si_7.98_Al_0.02_]­O_20_(OH)_4_, and the most widely used synthetic nanohectorite, laponite, (provided by BYK-Chemie GmbH), with chemical formula Na_0.7_(Si_8_Mg_5.5_Li_0.3_)­O_20_(OH)_4_. Manganese was introduced into these structures by cation-exchange reactions.

Ion-exchange processes were carried out on both montmorillonite and laponite. For each clay, 500 mL solutions of either Mn­(NO_3_)_2_ (Merck 99.9%) or MnCl_2_ (Merck 99.9%) were prepared at a concentration equivalent to 5 times their CEC. The first exchange cycle was performed by dispersing 3 g of clay in the as-prepared solution and stirring for 24 h. After that, samples were washed with deionized water and centrifuged at 5000 rpm for 15 min. The 24 h exchange process was repeated 3 more times.

Finally, the resulting powder was dried at 65 °C for 24 h. Specifically, one sample of Mn-exchanged montmorillonite was prepared using the Mn­(NO_3_)_2_ solution, while two samples of Mn-exchanged laponite were prepared using Mn­(NO_3_)_2_ and MnCl_2_. For clarity, the three used samples are named Mn-montmorillonite, Mn-Laponite1, and Mn-Laponite2, respectively.

### 2.2 Structural Characterization

Structural characterization was performed to confirm clay integrity after Mn exchange and to evaluate possible changes in basal spacing and layer organization. X-ray diffraction (XRD) patterns using a Bruker D8 Advance diffractometer with Bragg–Brentano geometry and a Cu *K*α anode operating at 40 kV and 25 mA. XRD patterns were obtained in the 3°–70° 2θ range with a 0.03° step and a 30 s accumulation time. Transmission electron microscopy (TEM) images were obtained in a JEOL JEM 1011 microscope.

### 2.3 Elemental Characterization

Direct solid analysis (DSA) of samples quantified by total-reflection X-ray (TXRF) was employed to quantify Mn associated with the solid phase. In contrast to conventional X-ray spectrometric methods, The DSA-TXRF enables the analysis of very small sample masses (typically <10 μg) and uses an adequate grinding, suspension, and internal standardization, providing bulk elemental concentrations. Notably, TXRF is not a surface-sensitive technique.^41^ A TXRF spectrometer model 8030c (FEI) was used for the analysis of Mn- exchanged clay solid samples. Measurements were conducted according to the manufacturer’s guidelines to ensure optimal excitation conditions and accurate spectral acquisition.

A pseudototal digestion was also performed on the samples to determine total elemental content. For that, 50 mg of clay was mineralized with 5 mL of HNO_3_ (Suprapur, 65%) and 5 mL of H_2_O_2_ (Suprapur, 30%) for 30 min at 80 °C on a heating plate. The solution was then filtered through 1–3 μm pore paper filters (Filter-lab, 1246) and diluted to a final volume of 50 mL with 2% HNO_3_ (Suprapur) and analyzed using an Inductively Coupled Plasma Optical Emission Spectroscopy (ICP-OES, iCAP Pro 7200 Plus, Thermo-Fisher Scientific), determining the total Mn loading.

### 2.4 Nutrient Release in Aqueous Solution

Release experiments were designed to evaluate the capacity of Mn-exchanged clays to provide a controlled and sustained Mn supply under alkaline conditions. A quantity of 4.8 mg of each clay was allowed to interact in a minimal saline medium (0.01 M ionic strength with KCl) and a Ca^2+^ concentration of 10^–3^ M at different pH values (5.5, 7.0, and 8.2) in closed plastic containers of 12 mL for 1, 3, 7, and 14 days, continuously shaken at room temperature and 90 cycles min^–1^. The presence of Ca^2+^ and low ionic strength conditions was selected to evaluate the stability and exchange behavior of Mn in Mn-exchanged clays under Ca-rich environments representative of alkaline soils. Three replicates per clay, pH, and time, together with two blank samples, were assayed, giving a total number of 180 samples. After the specified time, the samples were filtered through 1–3 μm pore paper filters (Filter-lab, 1246). Finally, the filtrate was diluted and acidified with HCl 1:1 (37%, Merck) for their analysis of total Mn concentration by Flame Atomic Absorption Spectrophotometry (AAS, PerkinElmer AAnalyst TM 800, Waltham, MA).

### 2.5 Plant Experiment

The plant experiment was designed to evaluate the effectiveness of Mn-exchanged clays as Mn fertilizers under alkaline conditions by releasing Mn into the nutrient solution and the ability of plants to efficiently uptake and translocate it. Application rates were defined to ensure comparable Mn input per plant across treatments, based on the total Mn content in the clays and on their maximum release behavior observed in the previous nutrient-released aqueous experiment. The experiment was conducted under climate controlled conditions in a growth chamber (Dycometal model typ CCK) with day/night temperatures of 23/18 °C, controlled humidity of 40–60%, and a light regime of 16h/day/8h/night. The growing media was a standard sand (CEN 196, grain size between 0.08 and 2 mm) mixed with the Mn-clay prepared by mixing 17.7 mg of Mn-laponites or 8.85 mg of Mn-montmorillonite with 80 g of the sand. The mixture was homogenized for 30 min in closed recipients in an orbital shaker. The quantity of each clay was calculated based on the Mn concentration, using the data obtained by the TXRF analysis (section 2.2) and the maximum Mn release determined in the nutrient release experiment (section 2.4), yielding an equivalent dose of 0.050 mg Mn/plant. Positive controls were prepared by mixing an equivalent amount of Mn as Mn-EDTA (Tradecorp Mn 13%) or MnSO_4_ (MnSO_4 ·_ H_2_O, Merck ≥ 98.0%) with the sand.

An experimental plant setup methodology similar to that described by López-Rayo et al. (2017)^40^ was used. For that, 160 mL polypropylene cylindrical vessels with three 2 cm-diameter holes at the bottom were used as pots. The vessels were first filled with 41 g of sand, then with 80 g of the sand-clay mixture, and finally completed with sand, resulting in a final sand weight of 227 g. By this arrangement, the clay was placed in the lower-middle part of the pots, allowing greater contact with the roots. For each treatment, 5 replicates (5 pots) were included, and two growing times (short-term: 4 weeks; long-term: 8 weeks) were evaluated, with individual pots assayed at each time.

A Mn-inefficient genotype of barley (*Hordeum vulgare* cv. Antonia), already described in the literature^42^ for its Mn sensitivity, was used to better observe the effect of the Mn treatments. Seeds were germinated by moistening in the dark for 24 h using Milli-Q type I ultrapure water with continuous aeration, and then, 2 seeds were placed in each pot, making a small hole in the top of the sand. The pots’ surfaces were covered with plastic film to conserve moisture and encourage germination. The humidity was controlled by weighing pots until 80% of the sand’s field capacity was reached. After 8 days, seedling development was observed, and only one seedling was retained. A Mn-free nutrient solution (López-Rayo et al., 2017) was added every 2 days, alternating with carbonated water, to maintain the pH of the growing media at 8.2. The composition of the Mn-free nutrient solution was: 0.2 mmol/L KH2PO4, 0.2 mmol/L K2SO4, 0.3 mmol/L MgSO4·7H2O, 0.3 mmol/L Mg­(NO3)­2·6H2O, 0.9 mmol/L Ca­(NO3)­2·4H2O, 0.6 mmol/L KNO3, 4 μmol/L Na-EDTA, 0.8 μmol/L Na2MoO4·2H2O, 0.7 μmol/L ZnCl2, 2 μM H3BO3, 1 μmol/L NiCl2·6H2O, 5 μmol/L Fe-EDTA.

Five pots per treatment were sampled at each sampling time (4 and 8 weeks); the shoots were harvested, washed sequentially with a solution containing distilled water, 0.01% Tween 80 (Sigma), and 0.1% HCl, then twice with distilled water, and finally dried on paper and weighed to determine the fresh weight.^43^ Finally, samples were placed in the forced-air oven (Memmert Loading Model 100–800) at 60 °C for 3 days to obtain the dry material. The wet sandy substrate from the pots was placed in plastic bags and stored at 4 °C for later analysis.

The dried plant material was weighed and milled in a porcelain mortar. The samples were placed in porcelain crucibles in a muffle furnace and submitted to 480 °C for 3 h. The ashes were digested with HNO_3_ (Suprapur 65%) on a heating plate at 80 °C for 30 min, filtered through a 20–25 μm pore-size paper filter, and diluted with Milli-Q water to reach a 2% acid media.

Water and DTPA extractions were performed to differentiate between immediately soluble and plant-available Mn fractions in the substrate. The sand substrate remaining in the pots was placed in 500 mL plastic bottles, to which 50 mL of Milli-Q water was added. They were placed on overturning shakers for 10 min, and the resulting solution was filtered through a 1–3 μm pore size filter (Filter-Lab 1246). A 1% acid matrix with HNO_3_ Suprapur (65%) was applied for further analysis of this soluble fraction. The extraction of the available fraction was then performed on the remaining substrate. For this, 120 mL of DTPA-TEA solution^44^ was added to each 500 mL bottle containing the substrate, and the bottles were placed in the overturning shaker for 15 min. The solutions were then filtered through a 1–3 μm pore-size filter (Filter-Lab 1246). Finally, 25 mL of this solution was taken and acidified to reach a 1% acid matrix with HNO_3_ Suprapur.

All plant-digested samples and soluble and available substrate extract samples were analyzed for their metal concentrations by Inductively Coupled Plasma Atomic Emission Spectroscopy (ICP-OES, Thermo Fisher Scientific, Waltham, MA).

### 2.6 Statistical Analysis

An analysis of variance was performed for all parameters, with a prior Levene test for homogeneity of variances, followed by a one-way analysis of variance (ANOVA) to identify differences among treatments. Either Duncan’s or Games-Howell post hoc test was applied, depending on whether variance homogeneity was met at a *p* = 0.05. The SPSS statistical software (version 26; SPSS Inc. Chicago, IL) was used.

## 3 Results

### 3.1 Structural Characterization and Chemical Composition of Clays

Montmorillonite is a swelling clay, formed by two siloxane tetrahedral sheets that sandwich an aluminum octahedral sheet, with a CEC = 79 mequiv/100 g. Isomorphic substitutions of Si^4+^ in the tetrahedral sheet and Al^3+^ in the octahedral one, by either Al^3+^ or Fe^2+^ and Mg^2+^, respectively, create a negative layer charge, which is compensated by interlayer exchangeable cations. Figure 1(a) compares the diffraction patterns of montmorillonite before and after exchange with Mn (Mn-montmorillonite). Montmorillonite exhibits characteristic general and basal reflections, with the main peaks assigned in the figure. Specifically, the general reflections or *hk* bands (with *l* = 0) are asymmetric and sawtooth-shaped due to the turbostratic nature of clays. Besides, symmetric basal (*00l*) reflections are assigned to the smectite-type crystalline structure. Finally, quartz impurities are also identified in the diffractogram. The particular strongest (001) peak centered at 2θ = 7.5 ° in the initial montmorillonite pattern corresponds to a basal spacing of ∼1.2 nm, associated with hydrated Na^+^ in the interlayer. The observed shift of the main (001) XRD peak to a lower 2θ value, which corresponds to a larger basal space of ∼1.4 nm, confirms the exchange of Na with Mn cations in the interlayer of montmorillonite.

**Figure 1:**
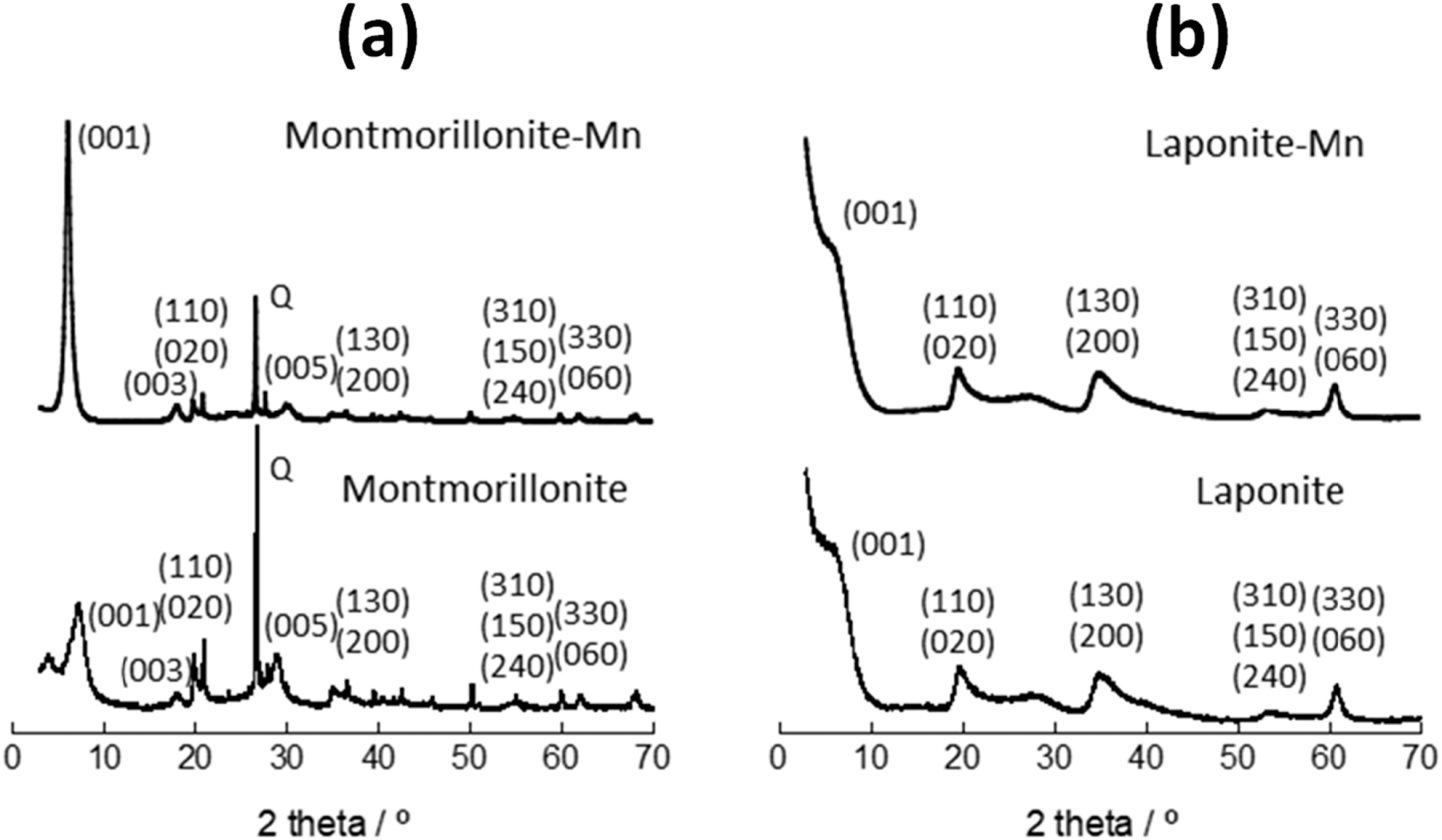
XRD patterns of the untreated (bottom) and Mn- exchanged (top) clays. (a) Montmorillonite. (b) Laponite.

Laponite is a disk-shaped commercial nanoclay with CEC = 70 mequiv/100 g and an average size of 25 nm. It consists of a single octahedral magnesia sheet sandwiched between two SiO_4_ based tetrahedral sheets. Isomorphic substitution of Mg^2+^ by Li^+^ within the octahedral layer generates a permanent negative layer charge. This charge is balanced by hydrated, exchangeable Na^+^ cations located in the interlayer space. Figure 1(b) shows the XRD patterns of laponite before and after exchange with Mn. Smectite reflections are present in both patterns. No evident changes in the basal spacing of Mn-laponite are observed, likely due to its nanometric particle size. In addition, because of their nanometric dimensions, Mn surface adsorption is expected to play a more significant role than in montmorillonite.

While XRD reveals structural changes such as interlayer expansion through shifts in the 001 reflection during cation exchange, TEM provides direct visualization, enabling accurate estimation of particle size and morphology in nanomaterials. Figure 2 presents a representative TEM image of the exchanged-nanoclay Mn-laponite. The image clearly highlights the layered structure, morphological uniformity, and nanometric dimensions of the particles, with diameters of ca. 25 nm.

**Figure 2:**
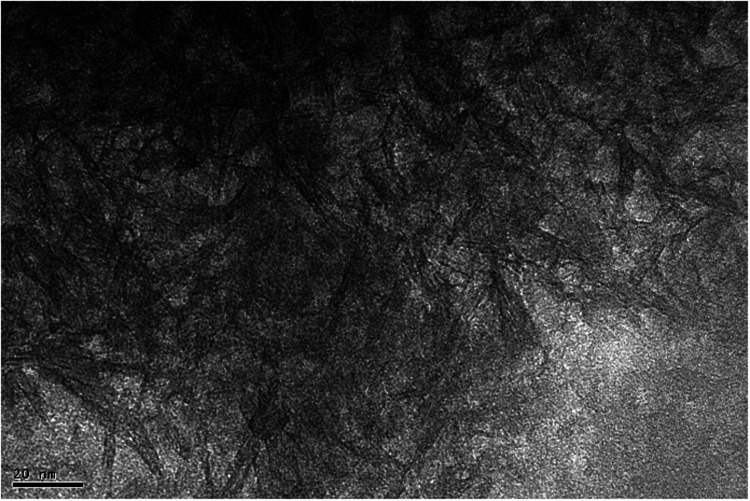
Representative TEM image of Mn-Laponite.

The CEC values obtained for montmorillonite and laponite (corresponding to 79 mequiv·100 g^–1^ and 70 mequiv·100 g^–1^, respectively) were used to estimate the maximum theoretical Mn content, by assuming Mn^2+^ incorporation within the clay interlayer, and considering the atomic mass of Mn and its divalent oxidation state. According to this, the maximum Mn loading in the laponite interlayer was calculated to be 19.2 mg·g^–1^, while for montmorillonite it was 21.7 mg·g^–1^. Then, TXRF and ICP-OES analyses were applied to compare the elemental content of the modified clays by quantitative experimental methods. The results obtained are shown in Table 1. These values were compared with the previous theoretical values calculated from CEC and stoichiometric composition. The Mn concentrations experimentally determined for both Mn-laponite1 and Mn-laponite2 by ICP-OES and TXRF were higher, with values close to 36 mg·g^–1^. This means an experimental Mn concentration 1.9-fold higher than the maximum Mn exchangable, an overestimation that could be attributed to the high specific surface area and nanometric particle size of laponite, which promotes the surface adsorption of Mn^2+^ ions beyond their structural incorporation into the clay matrix. This is in good agreement to the XRD pattern previously discussed.

**Table 1:** Elemental Composition of the Mn-Clays as Determined by Total-Reflection X-ray Fluorescence (TXRF) and Inductively Coupled Plasma Optical Emission Spectrometry (ICP-OES)^a^,^b^

mg/g clay				
clays	Mn	Al	Fe	Mg
ICP-OES				
Mn-Lap1	38.6 ± 0.1	5.58 ± 0.06	2.19 ± 0.26	146 ± 0.3
Mn-Lap2	38.0 ± 0.4	6.41 ± 0.53	2.34 ± 0.09	144 ± 0.5
Mn-Mont	21.5 ± 0.4	13.9 ± 0.45	51.1 ± 1.21	3.26 ± 0.04
TXRF				
Mn-Lap1	37.4 ± 0.1	nd	0.279 ± 0.005	37.4 ± 1.1
Mn-Lap2	36.2 ± 0.1	nd	0.466 ± 0.007	98.5 ± 3.4
Mn-Mont	26.2 ± 0.1	66.2 ± 1.7	22.6 ± 0.1	nd

a nd: Values under the limit of detection.

b Values correspond to experimentally measured concentrations. Values are means ± standard deviation (n = 3).

In contrast, the modified Mn-montmorillonite exhibited excellent agreement between the theoretical Mn calculated content (22.7 mg·g^–1^) and the experimental values obtained by ICP-OES (21.5 mg·g^–1^), and slightly lower than that obtained by TXRF (26.2 mg·g^–1^), indicating a more efficient and localized incorporation of Mn into the interlayer spaces of the smectite structure.

The concentration of other elements in the clays was analyzed, and the values were consistent with those expected for each clay type.

### 3.2 Nutrient Release in Aqueous Solution

Figure 3 shows the percentage of Mn released from the three Mn-exchanged clays (Mn-laponite1, Mn-laponite2, and Mn-montmorillonite) over 340 h under controlled pH conditions of 5.5, 7.0, and 8.2.

**Figure 3:**
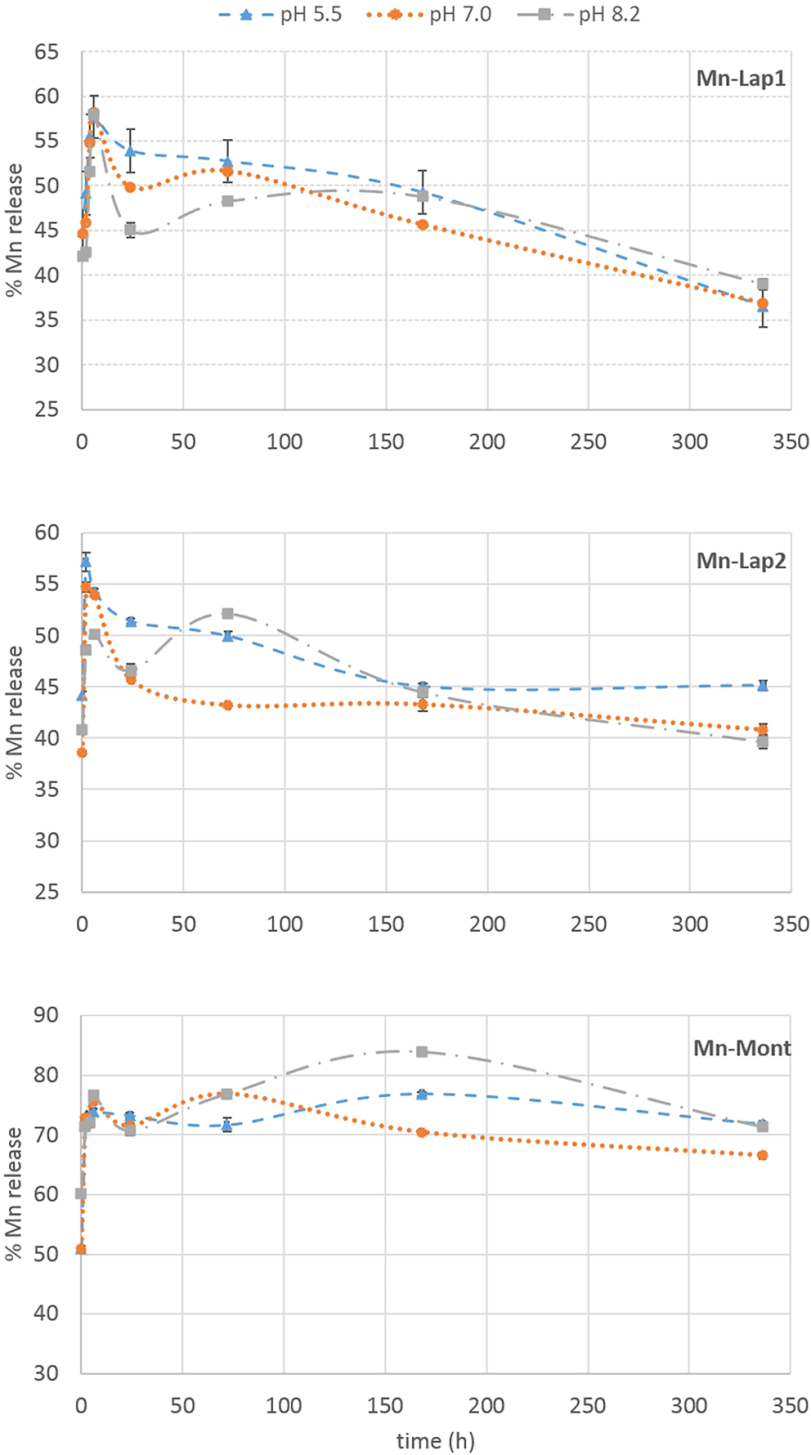
Percentage of Mn release from Mn-clays (calculated as the Mn concentration in solution relative to the total Mn content according to the theoretical Mn determined in section 3.2) at different pH levels (5.5, 7.0, and 8.2) as a function of time. The dots represent mean values, and the bars represent the standard error (SE) (*N* = 3).

The three pH conditions resulted in an initial rapid release of Mn within the first 10–24 h for Mn-laponite1, followed by a gradual decrease over time. At pH 5.5, 7.0 and 8.2, the release profiles exhibited an initial maximum of around 58%. After this initial peak, a noticeable decrease was already observed at 24h, particulary at pH 7.0 and 8.0. The amount of Mn released gradually decreased during the experiment, reaching around 40% at the last point for all pH levels.

A similar trend was observed for Mn-laponite2, although the release percentages were slightly lower overall. At pH 5.5 and 7.0, the initial Mn release was approximately 55%, whereas at pH 8.2 it was noticeably lower. After the initial peak within 10–24 h, all curves showed a progressive decline, stabilizing around 40% by 340 h. Compared with Mn-laponite1, the Mn-laponite2 curves demonstrated less dispersion between pH conditions during the later stages.

In contrast, Mn-montmorillonite exhibited substantially higher Mn release than the two Mn-laponite samples across all pH conditions. Initial values ranged from 70% to 75%, followed by a moderate secondary increase to 85% around 100–150h at pH 7.0 and 8.2. After this midterm maximum, Mn release gradually declined, ultimately stabilizing at around 70% after 340 h, regardless of pH. Although pH 7.0 consistently produced the highest values throughout the experiment, the three curves remained relatively close to one another compared with the Mn-laponite samples.

### 3.3 Effect of Mn-Exchanged Clays on Barley Plants Nutrition and Development

The dry weight (DW) of the plants was not significantly affected by the application of Mn-exchanged clays or conventional Mn fertilizer at the short-term of 4 weeks (Table 2). However, by 8 weeks term, the effect of the Mn-treatments became evident. Plants cultivated with Mn-laponite1 exhibited marginally diminished root and shoot DW in comparison to the Mn-deficient control. Conversely, all alternative Mn-treatments (Mn-laponite2, Mn-montmorillonite, MnSO_4_, and MnEDTA) yielded DW values that were statistically analogous to or exceeded those of the control, dependent on the specific analyzed tissue. Specifically, the Mn-laponite1 treatment resulted in reduced biomass accumulation compared to the untreated control at this later stage. In contrast, Mn-laponite2, Mn-montmorillonite, MnSO_4_, and MnEDTA did not differ from the control for shoot DW and showed similar or slightly reduced root DW.

**Table 2:** Dry Weight (DW, g/plant) of the Shoots and Roots of Barley Plants after Short- (4 weeks) and Long-Term (8 weeks) in Sand Substrate^a^

	4 weeks		8 weeks	
treatments	root (g/plant)	shoot (g/plant)	root (g/plant)	shoot (g/plant)
C-	0.16 ± 0.02 ^ns^	0.11 ± 0.03 ^ns^	0.65 ± 0.05 ^a^	0.54 ± 0.06 ^a^
Mn-Lap1	0.13 ± 0.03	0.087 ± 0.021	0.52 ± 0.12 ^b^	0.47 ± 0.06 ^b^
Mn-Lap2	0.14 ± 0.05	0.10 ± 0.02	0.43 ± 0.07 ^b^	0.56 ± 0.06 ^a^
Mn-Mont	0.12 ± 0.03	0.082 ± 0.012	0.42 ± 0.06 ^b^	0.57 ± 0.03 ^a^
MnSO_4_	0.14 ± 0.03	0.092 ± 0.021	0.45 ± 0.07 ^b^	0.59 ± 0.03 ^a^
MnEDTA	0.11 ± 0.06	0.081 ± 0.040	0.43 ± 0.05 ^b^	0.58 ± 0.03 ^a^

a Treatments Refer to Untreated Sand (C−), and Mn-Enriched Sand with Mn-Laponite1 (Mn-Lap1), Mn-Laponite2 (Mn-Lap2), Mn-Montmorillonite (Mn-Mont), MnSO_4_, and MnEDTA. Values are means ± standard deviation (*n* = 5). Different letters in the same column and organ indicates significant differences (*p* < 0.05)

Figure 4 compares the manganese (Mn) concentration in shoots and roots of barley plants after short-term (4 weeks) and long-term (8 weeks) treatments After 4 weeks, a marked increase in the concentrations of Mn was observed in the shoots and roots of plants submitted to Mn-laponite1 and Mn-laponite2 treatments, producing a clear Mn concentration increase in comparison to the Mn-deficient control (C−). The application of Mn-laponite treatments resulted in elevated Mn concentrations in shoots, with approximately 1.6-fold (Mn-laponite1) and 1.5-fold (Mn-laponite2) increases compared to deficient plants. Similarly, in root tissue, Mn concentrations increased by 2.3-fold and 1.9-fold, respectively. An intermediate response was observed with the Mn-montmorillonite, comparable to that with MnSO_4_. The significant enhancement observed with the Mn-laponites was analogous to the effect of MnEDTA, indicating that Mn-laponites are highly effective Mn sources during the initial 4 weeks of growth. After 8 weeks, Mn concentrations in the shoots increased relative to the initial sampling; however, no significant differences were observed among treatments or between treatments and the untreated control. However, in roots, Mn concentrations decreased from 4 to 8 weeks across treatments, including the Mn-laponites, suggesting partial translocation of Mn from roots to shoots and depletion of Mn available in the growth media as plants continued to develop. Notwithstanding this decline, Mn-laponites still exhibited higher Mn concentrations in the roots than in the untreated control and montmorillonite. The contrast between sampling times was most pronounced for the Mn-laponites and MnEDTA, reflecting their initially high Mn supply during the first growth period.

**Figure 4:**
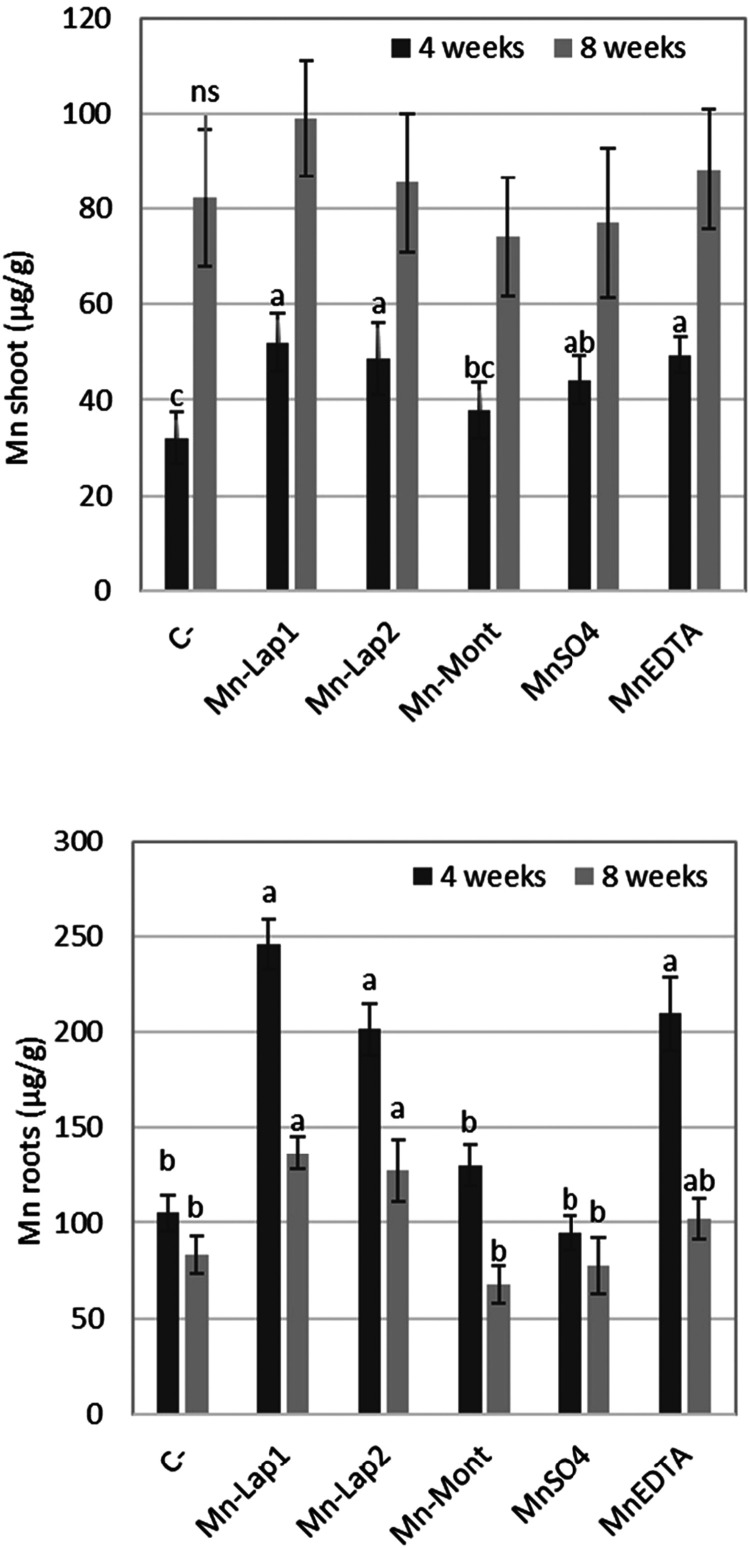
Manganese concentration of barley plants in the short- (4 weeks) and long-term (8 weeks) in shoots (upper part) and roots (lower part). Different letters indicate significant differences between treatments for shoots or roots at each time sampling, as determined by the Duncan or Games-Howell post hoc test (*P* < 0.05). *ns* denotes not significant differences. The error bars correspond to the SE (*N* = 5). Treatments refer to untreated sand (C−), and Mn-exchanged enriched sand with Mn-laponite1 (Mn-Lap1), Mn-laponite2 (Mn-Lap2), Mn-montmorillonite (Mn-Mont), MnSO_4_, and MnEDTA.

Analysis of Mn remaining in the soil after plant growth confirmed the distinct behavior of the Mn-clays and the comparison treatments (Figure 5). In the soluble fraction, slight differences were observed after a short-term of 4 weeks, with the Mn-laponite treatments showing high Mn levels, comparable to MnEDTA but statistically similar to Mn-montmorillonite, MnSO_4_, and the untreated control. Over the longer period (8 weeks), the same trend was maintened, with the Mn-Laponite treatments exihibiting the highest Mn concentrations among all treatments. In the available fraction, clearer differences emerged. Mn-laponite2 consistently produced the highest Mn reserves, with a 3.4-fold increase in available Mn compared to the control, highlighting its capacity to retain Mn in forms accessible for plant uptake over time. Mn-laponite1 showed lower Mn levels, yet still higher than those obtained with MnEDTA at both time points. In contrast, Mn-montmorillonite exhibited lower Mn retention in the available fraction, showing markedly weaker behavior than the laponites and similarly low as MnSO_4_. Interestingly, after 8 weeks, all Mn treatments retained slightly higher Mn reserves in the soil than the untreated medium (C−), indicating that even small differences reflected a consistent positive effect of Mn application.

**Figure 5:**
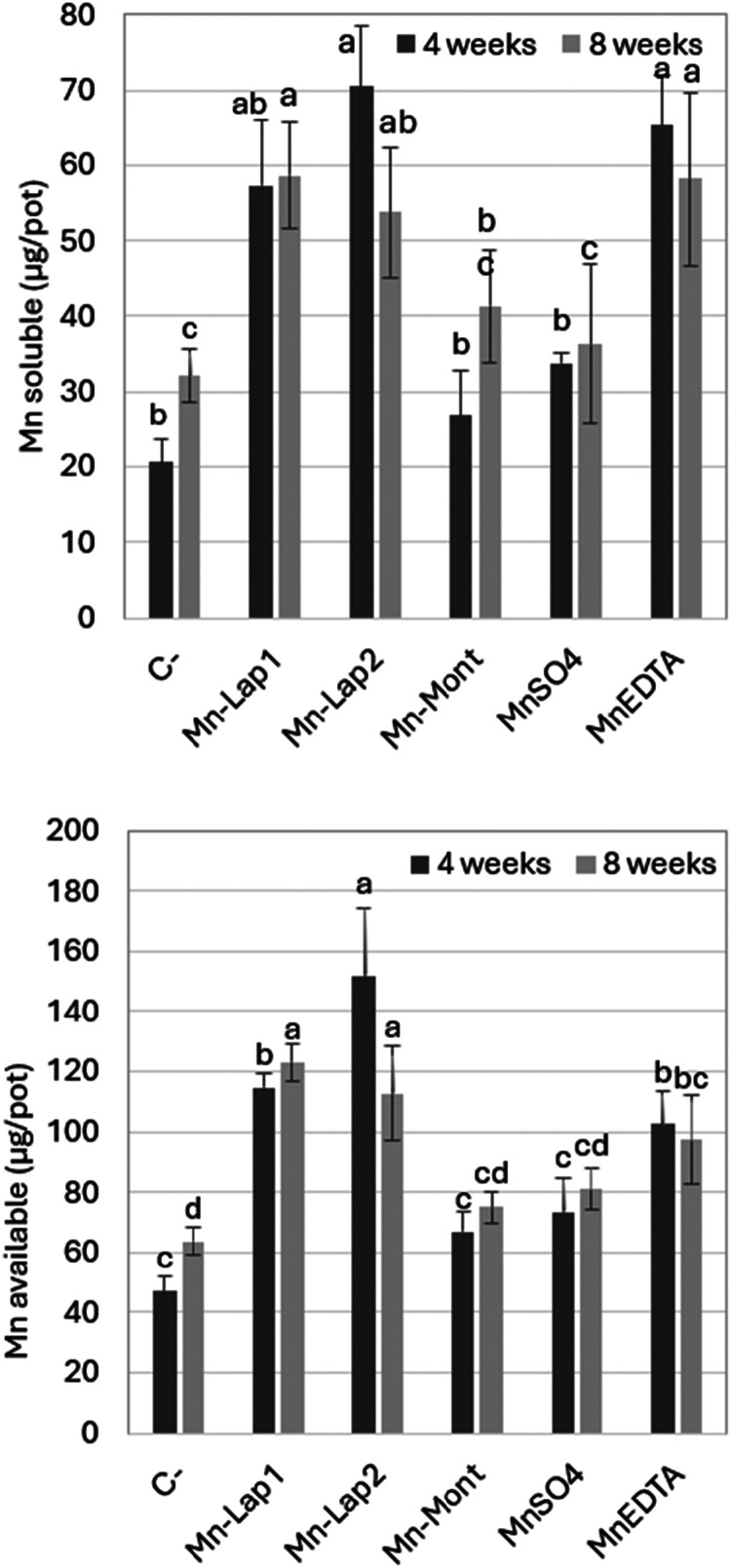
Manganese content expressed as μg Mn per pot determined in the soluble (a) and available (b) fractions in the substrate in the short- (4 weeks) and long-term (8 weeks) experiment. Different letters indicate significant differences between treatments for shoots or roots in each experiment, as determined by the Duncan or Games-Howell post hoc test (*P* < 0.05). *ns* denotes not significant differences. The error bars correspond to the SE (*N* = 5). Treatments refer to untreated sand (C−), and Mn-exchangeable enriched sand with Mn-laponite1 (Mn-Lap1), Mn-laponite2 (Mn-Lap2), Mn-montmorillonite (Mn-Mont), MnSO_4_, and MnEDTA.

## 4 Discussion

The results obtained reveal substantial differences in Mn release behavior between the Mn-exchanged laponites and montmorillonite, which can be attributed to variations in Mn incorporation efficiency arising from differences in clay properties, especially CEC and particle size.

In the Mn-laponite samples, the theoretical Mn content according to the CEC was 19.1 mg·g^–1^, whereas ICP-OES and TXRF analyses revealed significantly higher values (∼36 mg·g^–1^). This difference suggests that, in addition to the intended interlayer incorporation, additional surface adsorption or even partial precipitation of Mn species occurred during synthesis, processes favored by the high surface area and reactivity of laponite. Consequently, a substantial fraction of Mn is nonexchangeable and not governed by CEC constraints. This fact is in good agreement with the Mn release profile obtained in the nutrient release experiment. The rapid Mn release during the first 24 h can be associated with Mn species located at external or weakly coordinated sites. This behavior is consistent with laponite’s relatively low CEC, indicating that exchangeable positions do not exclusively govern Mn retention but are also influenced by heterogeneous interactions at particle surfaces and structural defects. After this initial surge, the release curves enter a slow, limited desorption stage dominated by more strongly retained interlayer Mn. The compact structure, low swelling capacity, and restricted interlayer expansion of laponite limit water penetration and hinder Mn diffusion from the interlayer region, leading to cumulative releases below ∼40% after 340–350 h. This behavior is consistent with the relatively limited pH sensitivity of the permanent layer charge and CEC of synthetic hectorite-type smectites in the near-neutral to mildly alkaline range, where only modest changes in exchange capacity are observed, and structural stability is maintained. Similar weak pH effects on sorption and release behavior at equilibrium have been reported for smectitic systems in which ion exchange dominates over pH-dependent edge reactions, supporting the interpretation that, in low-swelling synthetic clays, pH plays a secondary role once interlayer sites are saturated and equilibration is reached.

For Mn-exchanged montmorillonite, the theoretical Mn concentration estimated from CEC (21.7 mg·g^–1^) closely matched the value determined by ICP-OES (21.5 mg·g^–1^), indicating nearly quantitative occupation of exchange sites by Mn^2+^. The slightly higher Mn content measured by TXRF (26.2 mg·g^–1^) likely reflects the contribution of pre-existing structural Mn (which are coincident with the montmorillonite formulas) and minor amounts of nonexchangeable surface-bound Mn. Overall, these results indicate that Mn retention in montmorillonite is largely governed by cation exchange capacity,whereas in laponite significant Mn retention occurs beyond CEC constraints. In consequence, the Mn release properties are clearly different in the Mn-montmorillonite, reflecting their contrasting structural and physicochemical properties. The Mn-montmorillonite displays markedly higher and more sustained Mn release, reaching 70–85% at different pH values. The good agreement between the theoretical Mn loading and the Mn content determined by TXRF indicates efficient and homogeneous incorporation of Mn within the interlayer spaces of the smectite. The inherent high CEC of montmorillonite favors reversible Mn retention at exchange sites, while its pronounced swelling capacity facilitates water ingress and enhances Mn mobility within the interlayer domain (e.g., on cation mobility in smectites^45^). This combination of properties explains both the midterm increase in Mn release observed around 100–150 h and the consistently higher desorption compared with the Mn-laponites. The pH-dependent behavior of Mn-montmorillonite further underscores the role of structural charge and interlayer accessibility: under acidic conditions (pH 5.5), protonation decreases the net negative layer charge, weakening Mn retention and accelerating its release, whereas under alkaline conditions Mn release remains high, likely two possible factors: (i) competitive interactions with other cations (as is the case of the K^+^, Ca^2+^, and Na^+^ in the solution intentionally added to keep the ionic strength, to simulate alkaline conditions and to increase the pH by the NaOH addition, see section 2.4.) intensify competition for both exchange and edge sites, sustaining a significant Mn flux from the clay to the solution despite the strongly negative charge of the smectite structure; and (ii) increased accessibility of swollen interlayers (e.g., on pH-dependent cation exchange^46,47^ ). Altogether, these characteristics and the agronomic relevance of alkaline conditions in calcareous soils justify selecting pH 8.2 for the subsequent plant growth experiment.

Under calcareous conditions (pH 8.2), the lack of significant differences in dry weight (DW) at 4 weeks suggested that Mn release from both Mn-exchanged clays and conventional salts was sufficient to correct the initial deficiency rapidly. However, Mn deficiency in barley does not necessarily result in immediate biomass reductions under mild or latent conditions, as plants may continue to grow despite impaired physiological function. This is supported by studies showing that Mn deficiency in barley can remain latent, with no visible symptoms or growth reduction, while still causing significant impairments in photosynthetic processes.^42,48,49^

By 8 weeks, the generally similar or higher DW observed in all Mn-treated plants relative to the Mn-deficient control suggests that the total Mn supply and the timing of release from Mn-laponite2, Mn-montmorillonite, MnSO_4_, and MnEDTA adequately matched plant demand. This aligns with studies demonstrating that controlled-release or gradually available Mn sources can sustain growth while preventing toxicity during prolonged cultivation.^50–53^ The slight reduction in growth observed with Mn-laponite1 at 8 weeks may reflect either insufficient cumulative Mn release or, conversely, a localized oversupply that causes subtle toxicity or nutrient imbalances. Biomass reductions under high Mn availability or with more soluble Mn sources have been reported in wheat and citrus, even when tissue Mn concentrations remain within adequate ranges.

In calcareous soils, where Mn deficiency is common despite adequate total Mn,^54,55^ maintaining the Mn in exchangeable or complexed forms is relevant. The comparable DW values obtained with Mn-montmorillonite and MnEDTA are consistent with evidence that chelated or ligand-stabilized Mn sources keep Mn in solution and effectively alleviate Mn deficiency in high-pH media.^50,53^ Overall, the DW patterns support the concept that Mn-exchanged smectites, especially the Mn-montmorillonite, can serve as effective controlled-release Mn fertilizers at pH 8.2, provided that release kinetics are appropriately tuned to avoid insufficient or excessive Mn supply, both of which have been shown to suppress growth or increase oxidative stress in other Mn fertilization systems.^48,51,56^ The Mn concentration patterns in plant tissues and substrate further show that both Mn-exchanged smectites acted as effective short-term Mn sources under high-pH conditions, while also generating a persistent buffer of plant-available Mn in the growth medium.

The pronounced increase in Mn concentrations in roots and shoots at 4 weeks with both Mn-laponites, reaching levels comparable to MnEDTA, indicates that these clays released Mn quickly enough to overcome the well-known limitation of soil-applied Mn salts in alkaline substrates, where soluble Mn^2+^ is rapidly converted into Mn oxides of low plant availability.^55,57^ The similarity between Mn-montmorillonite and MnSO_4_ at 4 weeks suggests a more moderate release pattern, consistent with reports that many Mn fertilizers increase exchangeable Mn only transiently before repartitioning into less available pools.^48,49^ The convergence of shoot Mn concentrations across treatments at 8 weeks, together with the decline in root Mn, is consistent with the progressive depletion of labile Mn in the rhizosphere and partial translocation from roots to shoots as plants grow. This behavior has been related to the limited phloem mobility of Mn and its tight homeostatic regulation once adequate levels are achieved.^4^

Nevertheless, the fact that Mn-laponites maintained higher Mn concentrations in roots than both the control and Mn-montmorillonite may reflect a comparatively faster and less regulated Mn release in the rhizosphere, likely linked to the presence of surface-associated Mn species and their relatively rapid desorption or partial dissolution from the laponite structure, rather than rapid fixation into Mn oxides.^48^ Substrate fractionation data support this interpretation: Mn-laponite2, and to a lesser extent Mn-laponite1, generated the largest pools of the available Mn fraction while avoiding substantial increases in the soluble fraction, a pattern that may indicate a relatively labile and weakly retained Mn pool rather than a truly regulated release system.^49^ In contrast, Mn-montmorillonite and MnSO_4_ resulted in smaller immediate pools of available Mn, consistent with a stronger control of Mn retention and release dynamics. In the case of Mn-montmorillonite, this behavior is likely governed by interlayer cation exchange processes and diffusion constraints, which favor a more gradual and sustained Mn supply over time, as discussed for Mn dynamics in soils and amendments.^48,55^ This behavior is consistent with a more regulated release pattern in Mn-montmorillonite, compared with the comparatively less controlled Mn dynamics observed in Mn-laponites.

Taken together, the combined evidence from nutrient release, plant biomass, tissue Mn concentration, and substrate Mn dynamics indicates that Mn-montmorillonite functions as controlled-release Mn fertilizer in alkaline media. In contrast, Mn-laponite behaves more like a conventional, less efficient Mn source characterized by a rapid initial Mn supply. This rapid but nontoxic Mn supply from Mn-laponites during the first weeks is reflected in (i) elevated soluble/available Mn in the substrate, (ii) strongly increased Mn in roots and shoots, and (iii) improved early biomass, closely matching the performance of MnEDTA. However, this behavior is indicative of a high initial Mn availability rather than a regulated release mechanism and likely reflects the presence of readily labile Mn species, being more consistent with the rapid release patterns for many Mn formulations and geopolymers when not properly immobilized.^51^ In contrast, Mn-montmorillonite shows more moderate plant Mn and biomass responses together with lower immediate accumulation of available Mn in the substrate, consistent with a more regulated and gradual Mn supply. Rather than indicating lower efficiency, this behavior reflects stronger Mn retention within interlayer exchange sites and release governed by cation exchange and diffusion processes.

This behavior is particularly relevant because Mn deficiency is widespread and difficult to correct in calcareous and alkaline soils, where standard Mn fertilizers often yield erratic crop responses and poor efficiency unless pH is simultaneously lowered.^4,55^ Under these conditions, Mn-montmorillonite can be considered a more suitable candidate as controlled release Mn fertilizers, whereas Mn-laponites behave as a fast-release, availability driven Mn sources.

Overall, this study demonstrates that Mn-exchanged smectitic clays, especially Mn-montmorillonite, can act as effective control-release Mn fertilizers under alkaline conditions, in line with the main objectives and hypotheses. The synthesized clays displayed sufficient Mn loading, stability, and cation exchange capacity in calcium-rich, low-salinity environments, releasing Mn in a regulated manner rather than in a rapid, exhaustive pulse.

In an alkaline sand medium, Mn-montmorillonite efficiently corrected Mn deficiency and raised plant Mn concentrations. They maintained higher Mn levels in the plant-available substrate fraction over time, even when biomass responses were modest and likely limited by factors other than Mn. These results support the hypotheses that Mn-exchanged clays, especially Mn-montmorillonite, can provide a sustained Mn supply and improve Mn nutrition under alkaline conditions.

The contrasting behavior of Mn-laponites and Mn-montmorillonite confirms that smectite type is a key determinant of Mn-release dynamics and agronomic performance. Mn-laponites showed a less controlled release pattern. In contrast, Mn-montmorillonite behaved as a valid slow-release systems with a more gradual and persistent Mn supply. This validates the hypothesis that different Mn-smectites exhibit distinct fertilizer behaviors and highlights Mn-montmorillonite as a particularly promising material to address the current gap in efficient Mn fertilizers for alkaline growing media. The findings provide a proof-of-concept for using synthetic smectitic clays as tunable, controlled-release Mn fertilizers and highlight the need for further evaluation in real calcareous soils and field conditions.
